# Evolutionary and biogeographical implications of degraded LAGLIDADG endonuclease functionality and group I intron occurrence in stony corals (Scleractinia) and mushroom corals (Corallimorpharia)

**DOI:** 10.1371/journal.pone.0173734

**Published:** 2017-03-09

**Authors:** Juan Sebastián Celis, David R. Edgell, Björn Stelbrink, Daniel Wibberg, Torsten Hauffe, Jochen Blom, Jörn Kalinowski, Thomas Wilke

**Affiliations:** 1 Animal Ecology and Systematics, Justus Liebig University, Giessen, Hessen, Germany; 2 Corporation Center of Excellence in Marine Sciences CEMarin, Bogotá, Colombia; 3 Department of Biochemistry, School of Medicine and Dentistry, Western University, London, Ontario, Canada; 4 Department of Biochemistry, Institute for Genome Research and Systems Biology CeBiTec, Bielefeld University, Bielefeld, Germany; 5 Bioinformatics and Systems Biology, Justus Liebig University, Giessen, Germany; University of Toronto, CANADA

## Abstract

Group I introns and homing endonuclease genes (HEGs) are mobile genetic elements, capable of invading target sequences in intron-less genomes. LAGLIDADG HEGs are the largest family of endonucleases, playing a key role in the mobility of group I introns in a process known as ‘homing’. Group I introns and HEGs are rare in metazoans, and can be mainly found inserted in the *COXI* gene of some sponges and cnidarians, including stony corals (Scleractinia) and mushroom corals (Corallimorpharia). Vertical and horizontal intron transfer mechanisms have been proposed as explanations for intron occurrence in cnidarians. However, the central role of LAGLIDADG motifs in intron mobility mechanisms remains poorly understood. To resolve questions regarding the evolutionary origin and distribution of group I introns and HEGs in Scleractinia and Corallimorpharia, we examined intron/HEGs sequences within a comprehensive phylogenetic framework. Analyses of LAGLIDADG motif conservation showed a high degree of degradation in complex Scleractinia and Corallimorpharia. Moreover, the two motifs lack the respective acidic residues necessary for metal-ion binding and catalysis, potentially impairing horizontal intron mobility. In contrast, both motifs are highly conserved within robust Scleractinia, indicating a fully functional endonuclease capable of promoting horizontal intron transference. A higher rate of non-synonymous substitutions (K_a_) detected in the HEGs of complex Scleractinia and Corallimorpharia suggests degradation of the HEG, whereas lower K_a_ rates in robust Scleractinia are consistent with a scenario of purifying selection. Molecular-clock analyses and ancestral inference of intron type indicated an earlier intron insertion in complex Scleractinia and Corallimorpharia in comparison to robust Scleractinia. These findings suggest that the lack of horizontal intron transfers in the former two groups is related to an age-dependent degradation of the endonuclease activity. Moreover, they also explain the peculiar geographical patterns of introns in stony and mushroom corals.

## Introduction

Group I introns are self-splicing genetic elements with conserved secondary and tertiary structures that are involved in ribozyme activity at the RNA level [1]. Many group I introns also constitute mobile genetic elements at the DNA level due to mobility-promoting proteins, termed homing endonucleases (HEs), which are encoded by a homing endonuclease gene (HEG) inserted within the intron [2–5]. Currently, there are six known families of HEs [6, 7]. They are classified on the basis of conserved amino acid motifs that form the catalytic center or structural core of the enzyme, with the LAGLIDADG family being the largest and most diverse [4]. The intron/HEG association is a notable example of a composite mobile genetic element that has persisted by exploiting cellular DNA repair and recombination pathways to promote spread by a process known as ‘homing’ [8]. In the homing pathway, the intron-encoded HE generates a double-strand break in cognate genes that lack the intron, stimulating repair of the broken gene by using the intron-containing gene as template [2].

HEs tend to target conserved nucleotides that correspond to functionally critical amino acids of cellular genes, ensuring that a target site will be present in related genomes [9–11]. Moreover, HEs can tolerate nucleotide substitutions within their target site [5], accommodating genetic drift and natural variation in an apparent adaptation to enable efficient intron homing [2,12,13]. By inserting in phenotypically neutral locations within the intron (i.e., loop regions), HEGs may greatly reduce deleterious effects to the host [14]. Furthermore, the close association with introns allows the HEGs to spread horizontally through populations at high rates [13,15]. Likewise, the conserved amino acid motifs within HEs play a key role in horizontal transfer because they are directly involved in DNA endonuclease activity at target sequences [3,4]. The class-defining LAGLIDADG motif comprises a subunit interface with acidic residues (Aspartic [D, underlined] or Glutamic acid [E]), co-ordinating and positioning divalent metal ions necessary for hydrolysis of the DNA backbone [16,17]. Once all intron-less alleles are occupied, the potential for further intron-spread diminishes, leading to decreased selective pressure for maintaining endonuclease activity. Unless a population of intron-less alleles supports new insertions or the intron transposes to a new genomic location by reverse splicing or endonuclease-mediated cleavage [18–20], two outcomes due to loss of selection are possible: (i) degradation of the intron/HEG over evolutionary time, eventually leading to its deletion and/or (ii) a shift towards a RNA maturase that promotes intron splicing by a variety of mechanisms [21]. Based on these observations, and phylogenetic analyses of intron distribution in mitochondria, a cyclical life history of gain and loss was proposed for mobile group I introns [14,15]. Briefly, the cycle comprises horizontal intron invasion targeting intron-less alleles, fixation in the population, decreased selective pressure for maintaining endonuclease activity, degradation of the HEG, and complete intron/HEG deletion leading to target sequence re-establishment [13,15,22,23].

In metazoans, group I introns and HEGs are mainly known from sponges [24–26], placozoans [27], sea anemones [22,23,28] and scleractinian corals [29]. The order Scleractinia (stony corals) consists of two major phylogenetic groups, namely the ‘robust’ and the ‘complex’ clades [30,31] (hereafter referred to as robust Scleractinia and complex Scleractinia, respectively). However, there is still controversy regarding the monophyly of the Scleractinia, with the possibility of a sister group relationship between complex Scleractinia and Corallimorpharia [32–35]. The presence of a group I intron, interrupting the *COXI* (cytochrome c oxidase subunit I) gene, was previously reported by Fukami et al. in 20 species of robust Scleractinia [29]. Based on broadly similar topologies between host and intron-based phylogenies, they suggested that the observed pattern can be explained by vertical inheritance of the intron, although a sponge-coral horizontal transfer remained equally plausible as the origin of the intron in corals [29]. Furthermore, the authors proposed that the intron was originally transferred to robust Scleractinia from a fungal donor, even though the intron insertion site in the *COXI* gene differs between these two groups [29]. This contrasts with the general assumption that introns inserted in different positions are phylogenetically not closely related [36]. The exclusive vertical intron transfer proposed for robust Scleractinia is in disagreement with the widely accepted combined horizontal/vertical transfer mechanisms shown in sponges and sea anemones [22,23,25,26]. Moreover, the study of Fukami et al. did not include corals of complex Scleractinia, which contains some of the most important and widespread reef-building coral families [33].

Surprisingly, the functionality of the homing endonuclease assessed by the conservation degree of the LAGLIDADG motifs has not been considered previously in explaining occurrence of the intron in corals. Intron horizontal transfer requires an active endonuclease, since one function of intron-encoded LAGLIDADG enzymes is to serve as site-specific endonucleases within the mobility pathway of group I introns [37]. In comparison, inactive/degraded endonucleases constrain intron transfer to vertical inheritance [15]. Moreover, in contrast to sea anemones, the different stages of the intron cycle in corals [23], as well as the number of gains and/or losses within an absolute temporal framework, remain largely unknown. Finally, the effect of a restricted geographic occurrence of intron-containing taxa to the Indo-Pacific Ocean [29] has not been considered in previous studies of group I intron evolution in Scleractinia.

Given this lack of knowledge of intron/HEGs evolution in scleractinian corals and corallimorpharians, the general aim of this study is to unravel the role of LAGLIDADG endonucleases in the evolutionary history of group I intron occurrence in these groups. We used scleractinian corals and corallimorpharians as a model system because they commonly feature introns of potentially different origins [22,25], though being phylogenetically closely related [32,33]. Our specific aims are: 1) to identify the putative functionality of LAGLIDADG motifs and their role in the occurrence of group I introns; 2) to infer number and absolute times of gains or losses of introns within a molecular-clock framework, and 3) to assess the geographic distribution of intron-possessing species within an evolutionary context. We hypothesize that: (i) differences in LAGLIDADG motif conservation and hence homing endonuclease functionality among major groups of corals informs about horizontal/vertical intron transmission by comparing the putative LAGLIDADG motif composition between Scleractinia and Corallimorpharia; (ii) introns gained early during the evolutionary history of corals are more degraded than introns acquired more recently due to a longer time for degradation as part of the intron-cycle. Molecular-clock analyses in combination with information on LAGLIDADG motif composition and the inference of intron acquisition allow linking the time-dependent degradation of the LAGLIDADG motif, and (iii) introns obtained more recently show a more restricted geographical distribution than older introns. Biogeographic patterns of intron-containing taxa together with time-calibrated phylogenies and the LAGLIDADG motif assessment help explaining the intron occurrence over space and time.

By using a comprehensive phylogenetic framework, our results will likely provide insights into the occurrence of mitochondrial group I introns based on homing endonuclease functionality. Moreover, our approach has the potential of addressing evolutionary questions related to the evolutionary history of introns in other metazoans.

## Materials and methods

### *COXI* intron-exon sequence alignment and LAGLIDADG homing endonuclease homology validation

In order to retrieve the available homologous *COXI* intron sequences and their 5´ and 3´ flanking exons from Scleractinia and Corallimorpharia, a BLASTN [38,39] search was performed taking the *Porites rus* (complex Scleractinia) intron that we annotated previously [40], and the reported intron of *Physogyra lichtensteini* (robust Scleractinia) [29] as query sequences. This step was performed in an iterative procedure in which sequences identified during each step were used to refine the search [41]. We stopped the search when no new homologs were detected. By the time of searching (March 1st, 2016), 14 intron sequences for complex Scleractinia, 23 for robust Scleractinia and 11 for Corallimorpharia were retrieved (S1 Table). Using this methodology we retrieved only group I introns present in the *COXI* gene. Intron sequences belonging to complex Scleractinia lacked annotation of the HEG [hereafter referred to as LAGLIDADG Open Reading Frames (ORFs)]. Therefore the LAGLIDADG ORFs were annotated using the GenDB platform [42] and GLIMMER 3.0.2 [43]. We obtained information concerning intron presence/absence by performing a comprehensive literature and database search. All retrieved intron sequences were aligned in BioEdit version 7.2.5 [44] with the ClustalW algorithm [45] and default settings. Additionally, we aligned intron sequences found in complex Scleractinia using information on the intron secondary structure (S1 Fig). Intron insertion sites were defined following the guidelines proposed by the Human Genome Variation Society [46], using the reference human *COXI* gene (Cambridge human mtDNA sequence; GenBank accession number NC_012920.1). We evaluated the conservation of potential target sequences for intron-encoded HEs by aligning selected *COXI* exon sequences from Scleractinia and Corallimorpharia. Taxa were chosen to represent the *COXI* intron insertion sites known among Scleractinia (S2 Fig).

Protein structure modeling of the intron-encoded LAGLIDADG homing endonuclease (LHE) found in *P*. *rus* (complex Scleractinia), *P*. *lichtensteini* (robust Scleractinia) and *Ricordea florida* (Corallimorpharia) was performed in Phyre2 [47] in order to validate their similarity to known LHEs. The generated LHE structure models were constructed based on the experimentally validated crystal structure of the I-SmaMI LHE found in the fungus *Sordaria macrospora* (Molecular Modeling Data Base ID: 123349). Both the predicted secondary structures and the known and predicted secondary structure of *S*. *macrospora* are used in conjunction with the sequence information in generating the alignment. By this means, is possible to identify the two LAGLIDADG motifs as part of the two core α helices, consistent with the accepted canonical LHE structure [7].

### Synonymous vs. non-synonymous ratios (K_a_/K_s_) and sequence logo representation of amino acid conservation

To determine whether the LAGLIDADG ORFs of Scleractinia and Corallimorpharia are under different types of selection, ratios of synonymous versus non-synonymous substitutions (K_a_/K_s_) were calculated following Emblem et al. [23]. Thereby, a K_a_/K_s_ ratio >1 indicates substitutions that more frequently result in a change of amino acid identity, consistent with positive selection. K_a_/K_s_ ratios <1 indicate substitutions that retain amino acid identity, as expected for purifying (negative) selection, while a K_a_/K_s_ ratio of ~1 reflects neutral evolution [23].

The predicted amino acid sequences encoded by each of the LAGLIDADG ORFs of 23 robust Scleractinia, 14 complex Scleractinia, and 11 Corallimorpharia species were reverse translated to generate nucleotide sequence alignments in MEGA version 7 [48]. We excluded codons containing alignment gaps in any sequence from subsequent analyses. The final HEG alignment lengths were 925, 753, and 453 bp for robust Scleractinia, complex Scleractinia, and Corallimorpharia, respectively. K_a_/K_s_ calculations of the HEG alignments were performed for each alignment in MEGA using the mold-yeast mitochondrial genetic code [23,32]. Substitutions rates were calculated using the Nei-Gojobori nucleotide diversity estimates [49] and applying the Jukes-Cantor correction for multiple substitutions. Pairwise comparisons of the HEG were plotted using a custom script for the R statistical environment v.3.2.1 [50]. A one-sided codon-based Z-test of selection [48], as implemented in MEGA, was used to test for purifying selection acting on the translated LAGLIDADG ORFs (H_0_: K_a_ = K_s_ vs. H_A_: K_a_<K_s_) with variance estimated with 1000 bootstrap replicates. This analysis was performed by averaging over all sequence pairs of the aligned HEs datasets of robust Scleractinia, complex Scleractinia, and Corallimorpharia (Table 1). The K_a_/K_s_ ratios of the C*OXI* Folmer region [51], a 658 bp fragment widely used for phylogenetic inference, of the intron-containing taxa were compared to the ratios calculated for the LAGLIDADG ORFs in order to test for relaxed purifying selection (S3 Fig). Sequence logo representation of amino acid conservation in the LAGLIDADG motifs was generated for complex Scleractinia, robust Scleractinia, and Corallimorpharia endonucleases using WebLogo [52].

**Table 1:** Codon-based Z-test of purifying selection in LAGLIDADG ORFs of Scleractinia and Corallimorpharia.

	Number of LAGLIDADG sequences	Number of positions in the final dataset	P	Z-statistic
Robust Scleractinia	23	307	<0.001	4.466
Complex Scleractinia	14	249	<0.001	7.296
Corallimopharia	11	150	<0.001	3.507

The probability *P* of rejecting the null hypothesis of strict neutrality (K_a_ = K_s_) in favor of the alternative hypothesis (K_a_<K_s_) is shown. *P* values less than 0.05 are considered significant at the α 5% level. The test statistic z (K_s_-K_a_) is shown in the Stat column. K_s_ and K_a_ are the numbers of synonymous and nonsynonymous substitutions per site, respectively.

### Estimation of divergence times and ancestral intron types

For the molecular-clock analysis, we used a 171-taxon dataset of two mitochondrial genes; *COXI* and *cyt b*, available from GenBank (see S2 Table for accession numbers). Mitochondrial genes have been widely used for phylogenetic studies in corals [29,32,33,53] given their low substitution rates in this group [33]. The dataset included 149 of the c. 780 zooxanthellate scleractinian species (19%), 12 corallimorpharian and 10 outgroup taxa belonging to the Actiniaria, Antipatharia, Zoantharia, Octocorallia, and Porifera.

We removed the intron-containing species *Caulastraea echinulata* (robust Scleractinia) from the molecular-clock analysis due to the lack of respective *cyt b* sequences in GenBank. The concatenated dataset had a total length of 1349 bp. Divergence times were inferred in BEAST v. 1.8.2 [54]. An initial uncorrelated relaxed-clock analysis using substitution models for *COXI* and *cyt b* (jModelTest, 24 candidate substitutions models, best AIC: GTR+I+G) [55] and relative rates was performed in order to retrieve the topology. In the final analysis, a set of fossil calibration points was used as suggested by Simpson et al. [53]. Given the uncertainty of these calibration points, Simpson et al. used uniform priors for the node calibration. However, since such priors represent hard constraints and often result in initial posterior probabilities to be zero, we here used normal distribution priors. Moreover, three out of the eleven suggested fossil calibration points had to be excluded due to a slightly different sampling design. Thus, the final analyses included the following eight fossil calibration points used by Simpson et al. [53]: (1) MRCA Scleractinia, 245–271 My (BEAST settings: mean = 258, standard deviation = 6.6); (2) *Isopora*–*Acropora*, 5.3–15 My (mean = 10.15, SD = 2.5); (3) *Acropora*–*Montipora*, 37.2–67.7 My (mean = 52.45, SD = 7.8); (4) *Acropora*–*Stephanocoenia*, 63.4–90.1 My (mean = 76.75, SD = 6.8); (5); *Lobophyllia*–*Symphyllia*, 19–31.3 My (mean = 25.15, SD = 3.1); (6) *Pocillopora*–*Seriatopora*, 28.4–42.7 My (mean = 35.55, SD = 3.7); (7) *Echinopora*–*Oulophyllia*, 21.9–49.3 My (mean = 35.6, SD = 7.0); and (8) *Cladocora*–*Hydnophora* 100–183.3 My (mean = 141.65, SD = 21.2). Two replicates were run on the CIPRES Science Gateway web portal [56] using the following settings: ngen = 100,000,000; samplefreq = 5,000; birth-death (BD) model. Log files were visualized in Tracer v. 1.5 [57] for congruency and combined in LogCombiner v. 1.8.2 (BEAST package; 50% burnin). TreeAnnotator v. 1.8.2 (BEAST package; no additional burn-in) was used for combining the tree files and for identifying the maximum clade credibility (MCC) tree.

In order to infer intron ancestral states and frequency of gains and/or losses, ancestral inference of intron-type was performed employing stochastic character mapping [58] available in the package phytools version 0.5.10 [59] for R. This method can handle uncertainty of intron presence by permitting the input of a matrix of prior probabilities of intron state at the tips of the phylogeny. The analysis used the *COXI/cyt b* topology obtained from the divergence time inference. We defined five character states based on intron presence/absence and intron type: 1) intron absence; 2) intron missing information; 3) intron 720; 4) intron 884; 5) intron 867. In the case where intron presence/absence was unknown (i.e., missing) the character state was defined by a uniform prior. This approach not only infers ancestral states at internal nodes but also provides posterior probabilities of intron state for tips with unknown intron presence/absence [59].

## Results

### Group I introns in the *COXI* gene of Scleractinia and Corallimorpharia

To gain insight into the evolution of group I introns interrupting the *COXI* gene of Scleractinia and Corallimorpharia, we assembled a dataset comprising 48 introns in total. This dataset included the annotated group I intron of *P*. *rus* (complex Scleractinia) [40], introns and HEGs previously identified [28,29,32], and introns that we identified through database searches. Besides these 11 Corallimorpharia, 14 complex Scleractinia, and 23 robust Scleractinia, we also identified 19 intron-containing taxa belonging to Actiniaria, Antipatharia, Zoantharia, and Porifera. We utilized the latter taxa as outgroups in the time-calibrated phylogeny (see below).

**Fig 1:**
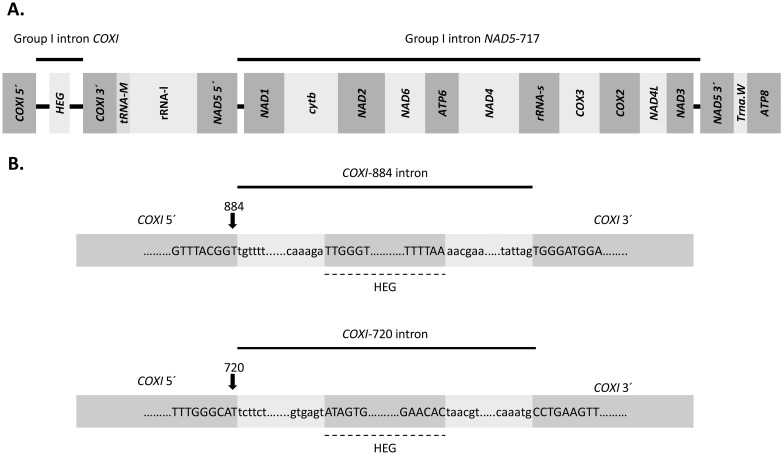
Mitochondrial gene content and schematic representation of group I intron and homing endonuclease genes found in Scleractinia. (A) Schematic map of the *P*. *rus* (complex Scleractinia) mitochondrial genome showing gene content and group I introns inserted in the *COXI* and *NAD5 genes*. The width of each box is proportional to gene size. (B) Representation of intron 884 (complex Scleractinia and Corallimorpharia) and intron 720 (robust Scleractinia) showing 5´and 3´ exons, and the putative homing endonuclease gene (HEG) of the LAGLIDADG family. The black arrows indicate intron insertion position in the *COXI* gene.

According to the established intron insertion site nomenclature based on the human mt genome [46], we defined two different introns inserted at the 3´ ends of positions 720 and 884 (hereafter referred to as intron 720 and intron 884, respectively) (Fig 1A and 1B, and see S2 Fig). Intron 720 was found in robust Scleractinia and *Plakinastrella* cf. *onkodes* (Porifera), and intron 884 in complex Scleractinia and Corallimorpharia. One additional intron type inserted at the 3´ end of position 867 was identified in *Savalia savaglia* (Zoantharia). Notably, we found differences regarding mean intron sizes of 1086±24 (standard deviation), 967±7 and 1203±28 base pairs (bp) for robust Scleractinia, complex Scleractinia, and Corallimorpharia, respectively. The difference in mean size obeys to a variable LAGLIDADG ORF size among the three groups, with the longest ORFs found in robust Scleractinia (mean size 962±31 bp), whereas complex Scleractinia and Corallimorpharia displayed shorter ORFs (755±1 and 778±207 bp, respectively). The alignment of the exonic regions flanking introns 720 and 884 of both intron-containing and intron-less taxa showed that the potential target sequences for the LAGLIDADG endonucleases are highly conserved in intron-less taxa (S2 Fig).

**Fig 2:**
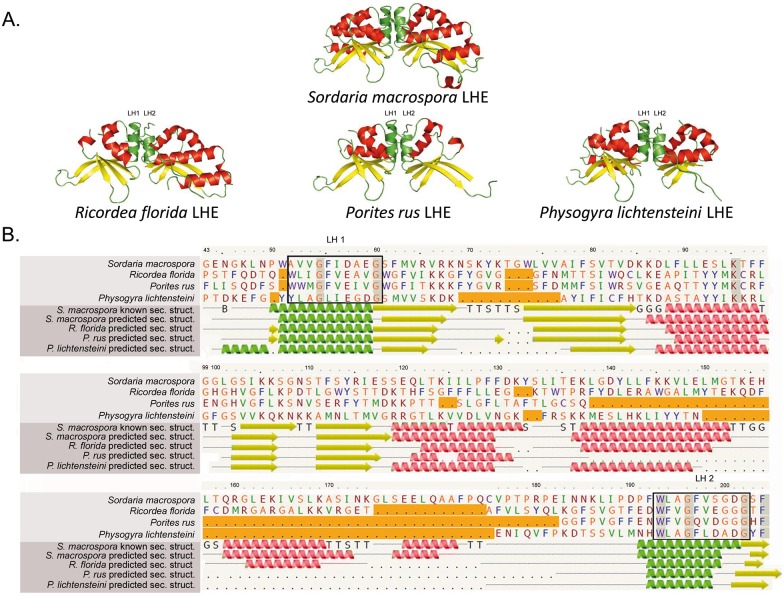
Predicted secondary structures and alignment of LAGLIDADG Homing Endonucleases (LHEs) found in Corallimorpharia and Scleractinia. (A) The experimentally validated LHE structure of the fungus *S*. *macrospora* shows the core elements being characteristic for canonical homing endonucleases. The predicted models of the LHEs found in *R*. *florida* (Corallimorpharia), *P*. *rus* (complex Scleractinia) and *P*. *lichtensteini* (robust Scleractinia) follow the general arrangement of helices, sheets and coils as in *S*. *macrospora*. The catalytic LAGLIDADG structures are represented by green helices (LH1 and LH2); red helices and yellow arrows depict the core α helices and the β sheets, respectively. (B) Alignment of the LHE based on residue similarity and secondary structure. The upper rows display the LHE amino acid sequences with orange and gray boxes representing deletions relative to *S*. *macrospora* and identical residues in the alignment, respectively. Lower rows show the known LHE structure of *S*. *macrospora* and the predicted secondary structures of the LHE analyzed. B, T, S and G in the *S*. *macrospora* row represent residues in isolated β-bridge, hydrogen bonded turn, bend and 3-turn helix (3_10_ helix), respectively.

Modeled structures of selected LHEs found in both complex and robust Scleractinia and Corallimorpharia are consistent with the experimentally determined LHE protein structure of the fungus *S*. *macrospora*. Thus, the two LAGLIDADG motifs were placed in the two central helices at the protein’s domain interface (Fig 2A). Likewise, the alignment of the LHEs of *P*. *rus*, *P*. *lichtensteini* and *R*. *florida*, based on residue similarity and predicted secondary structure is consistent with the known crystal structure of the LHE of *S*. *macrospora* (Fig 2B).

### LAGLIDADG motif conservation and evolutionary constrains on endonucleases in Scleractinia and Corallimorpharia

**Fig 3:**
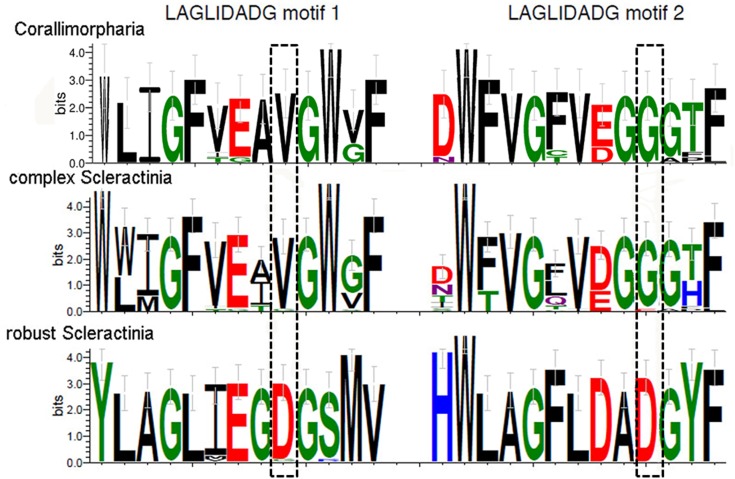
Sequence logo of the two LAGLIDADG motifs found in Corallimorpharia, complex Scleractinia and robust Scleractinia. All homing endonucleases have two LAGLIDADG motifs. Both motifs in complex Scleractinia are lacking the conserved acidic residues (D or E, highlighted by the dashed boxes) necessary for metal-ion binding and catalysis. Amino acids are color-coded according to chemical properties (blue, basic; red, acidic; green, polar; black, hydrophobic). Information content at each position (in bits) is represented by the height of the amino acid and its estimated variance is shown by the error bar. A score of 4.2 bits corresponds to high conservation, while a score of 0 corresponds to low conservation.

The sizes of the coding regions are consistent with single-chain monomeric LAGLIDADG enzymes. In total, we identified two LAGLIDADG motifs in all examined sequences (Fig 3 and S4 Fig). However, endonucleases found in complex Scleractinia are highly diverse. Moreover, both motifs lack the conserved acidic residues (D or E, highlighted by the dashed boxes in Fig 3) necessary for metal-ion binding and catalysis. In contrast, these residues were strictly conserved in LAGLIDADG ORFs found in robust Scleractinia. A number of the ORFs present in Corallimorpharia also lacked the metal-binding residues in the LAGLIDADG motifs.

**Fig 4:**
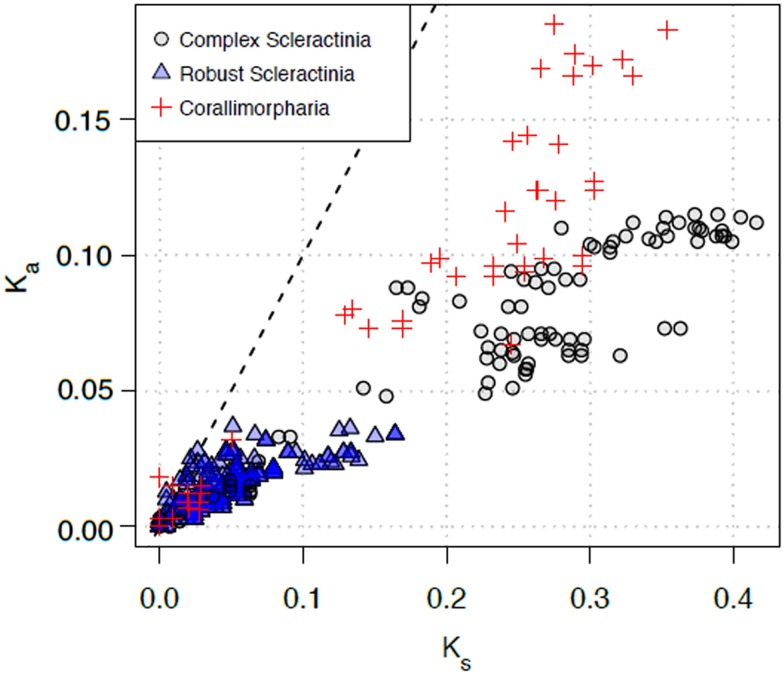
Evolutionary rates of homing endonucleases in complex Scleractinia, robust Scleractinia, and Corallimorpharia. The rate of nucleotide substitutions causing amino acid changes (K_a_) is plotted relative to substitutions at silent sites (K_s_) for each region and for all pairwise comparisons. Symbols represent K_a_/K_s_ ratios calculated for HEG. The dotted line indicates the theoretical expectation of neutral evolution (K_a_/K_s_ = 1). The area below the dotted line represents purifying selection (K_a_<K_s_).

To assess the nature of the selective forces acting on HEGs inserted in the intron of Scleractinia and Corallimorpharia, we estimated the rate of nucleotide substitutions at non-synonymous (K_a_) relative to synonymous sites (K_s_). Pairwise comparisons of HEGs sequences of complex Scleractinia displayed K_s_ rates between 0.007 and 0.416. The rates for robust Scleractinia ranged from 0 to 0.164, while HEGs within the Corallimorpharia showed K_s_ rates ranging from 0 to 0.354. In contrast, K_a_ rates in complex Scleractinia and Corallimorpharia displayed rates varying from 0.010 to 0.115 and 0 to 0.185, respectively; whereas robust Scleractinia showed considerably lower rates, ranging from 0 to 0.034 (Fig 4). In general, the K_a_/K_s_ ratios for Scleractinia and Corallimorpharia are smaller than 1, indicating purifying selection (Fig 4). The one-sided codon-based Z-test reaffirmed that the LAGLIDADG ORFs of Scleractinia and Corallimorpharia are under purifying selection, rejecting the null hypothesis of strict neutrality (K_a_/K_s_ = 1) in favor of the alternative hypothesis (K_a_/K_s_ <1) (p<0.05; Table 1). Finally, K_s_ rates calculated for the Folmer region of the *COXI* gene exhibited values ranging from 0 to 0.461 and K_a_ rates smaller than 0.034 (S3 Fig), with K_a_/K_s_ ratios below 1, indicating that the *COXI* gene is also under purifying selection. As K_a_ rates in complex Scleractinia and Corallimorpharia seem to be at least one order of magnitude higher than the rates in robust Scleractinia, HEG might be under relaxed purifying selection in the former two groups and under stronger purifying selection in the latter group.

### Intron distribution, molecular-clock analyses, and ancestral inference of intron types

**Fig 5:**
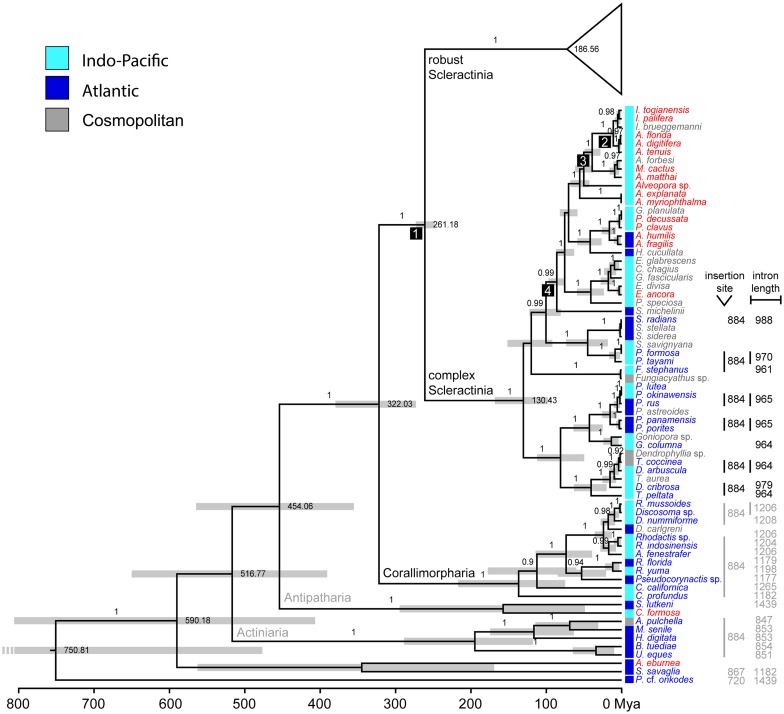
Molecular-clock analysis of major cnidarian groups and complex Scleractinia. A set of eight fossil calibration points was used as suggested by Simpson et al. [53]. Nodes used for calibration are denoted with numbered black boxes referring to the node numbers used in the Methods section. Posterior probabilities are indicated at branches. Bars at nodes indicate 95% credibility intervals. Taxa are color-coded according to the absence (red) or presence (blue) of an intron or whether no information is available (gray).The order Scleractinia is a highly supported monophyletic group, with the order Corallimorpharia being its sister group.

**Fig 6:**
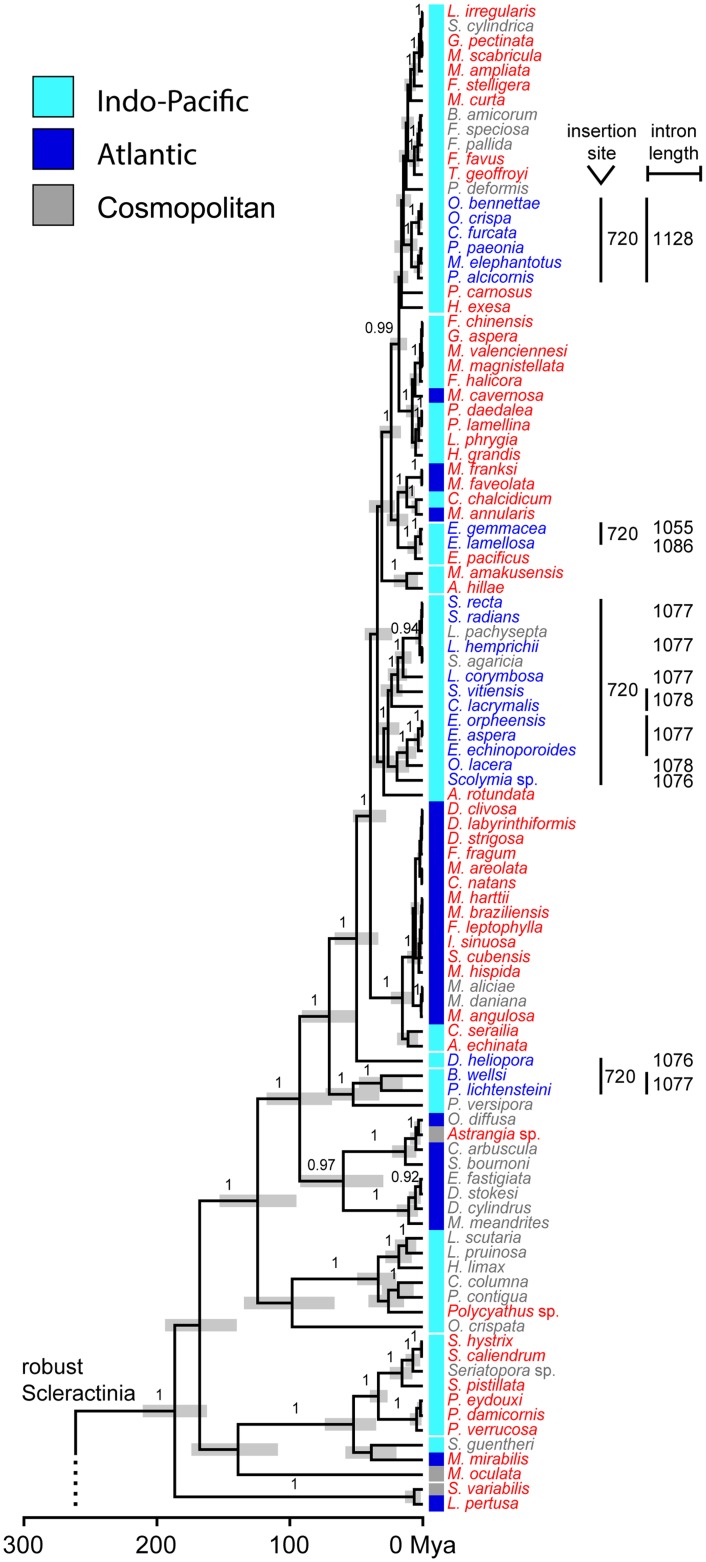
Molecular-clock analysis of robust Scleractinia. A set of eight fossil calibration points was used as suggested by Simpson et al. [53]. Posterior probabilities are placed on branches. Bars in nodes indicate 95% credibility intervals. Taxa are color-coded according to the absence (red) or presence (blue) of an intron or whether no information is available (gray).

Phylogenetic reconstructions using Bayesian inference (BEAST; relaxed-clock model) showed strong support for most scleractinian and corallimorpharian genera (Figs 5 and 6). The Scleractinia is a highly supported monophyletic group (posterior probability, pp: 1.0), with the Corallimorpharia (pp: 1.0) being its sister group (pp: 1.0). According to the time-calibrated phylogeny, the split of robust and complex Scleractinia occurred about 260 million years ago (mya) (248.01–273.19 mya, 95% highest posterior density, HPD) and the split of Corallimorpharia/Scleractinia about 320 mya (272.92–379.73 mya; Fig 5). Our results also showed that intron 720 is found in 22 taxa of robust Scleractinia and in Porifera, whereas intron 884 is present in 14 and 11 taxa of complex Scleractinia and Corallimorpharia, respectively, as well as in the outgroup taxa belonging to the Actiniaria and Antipatharia. Intron 867 was only found in the outgroup species *S*. *savaglia* (Zoantharia).

Within robust Scleractinia, intron 720 was found in four independent clusters and one lineage. In contrast, taxa containing intron 884 appear to form a single cluster within the complex Scleractinia. Moreover, the Corallimorpharia and the intron-containing outgroup taxa all contain intron 884, except for *P*. cf. *onkodes* (Porifera), which contains intron 720 (Fig 5). We also found that taxa possessing intron 720 are restricted to the Indo-Pacific Ocean, while intron 884 occurs in taxa inhabiting both Indo-Pacific and Atlantic/Mediterranean waters (Figs 5 and 6).

**Fig 7:**
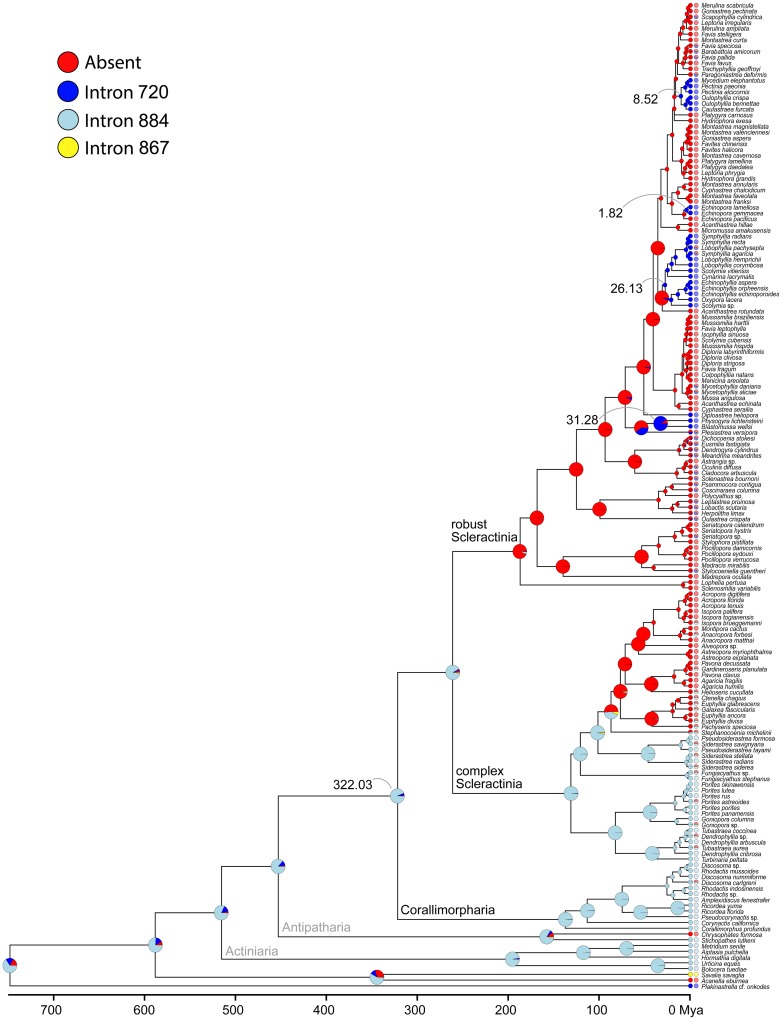
Bayesian stochastic character mapping of intron type in major cnidarian groups. The translucent pie charts portray the prior information about intron type used for the analysis. Single colored circles indicate known intron presence/absence, whereas in the case of unknown intron state proportions of pie charts display the respective prior probability. Solid colored pie charts show inferred posterior probabilities of intron state at internal nodes and tips.

The four intron 720 containing clusters were estimated to have a mean crown age of 1.82 (0.09–4.30), 8.52 (4.04–13.82), 26.13 (17.15–35.35), and 31.28 (15.25–48.04) my, with the respective nodes being highly supported (pp: all 1.0) (Fig 6). The robust Scleractinia has an approximate age of 185 my, with a high posterior probability (0.85) that the most recent common ancestor contained no intron. In contrast, the time of acquisition of intron 884 clearly predates the split between Scleractinia and Corallimorpharia c. 322 mya (Fig 7). However, intron 884 acquisitions and timing in Corallimorpharia remain elusive.

## Discussion

This study attempts to explore the pivotal role of LAGLIDADG motifs in the intron/HEG distribution in the closely related cnidarian orders Scleractinia and Corallimorpharia. Logo alignment of the HEGs, inserted both in intron 720 and 884, showed a degradation of the LAGLIDADG motifs together with a lack of acidic residues in complex Scleractinia and in Corallimorpharia. This suggests that the endonuclease is degraded, implying an impairment of its enzymatic activity. In contrast, the motifs were highly conserved in robust Scleractinia, consistent with a putatively functional endonuclease. Molecular-clock analyses and inference of ancestral intron types suggested at least five independent intron gains in robust Scleractinia, whereas complex Scleractinia and Corallimorpharia inherited their intron from a common ancestor shared by both groups. In addition, an earlier intron gain was estimated for complex Scleractinia and Corallimorpharia (>322.03 mya) compared to robust Scleractinia (31.28–1.82 mya). The longer presence of intron 884 in complex Scleractinia and Corallimorpharia might be related to the degradation of the homing endonuclease gene, consistent with the intron cycle hypothesis. This is further supported by the higher K_a_ rates detected for the endonuclease in complex Scleractinia and Corallimorpharia than in robust Scleractinia, suggesting that the LAGLIDADG endonuclease is under a relaxed purifying selection in the former two groups, allowing higher rates of amino acid replacements. Finally, we noticed a clear geographic restriction of intron 720 to taxa inhabiting the Indo-Pacific Ocean, while taxa containing intron 884 are cosmopolitan species.

### The LAGLIDADG motif as a major driver of group I intron transfer in Scleractinia and Corallimorpharia

Intron-encoded LAGLIDADG enzymes function primarily as site-specific endonucleases within the mobility pathway of group I introns. LAGLIDADG proteins can also act as RNA maturase by binding to and stabilizing the RNA secondary structure of the group I intron and thus promoting efficient RNA splicing. These two activities are not mutually exclusive, as some LAGLIDADG enzymes promote both mobility and splicing [4]. It has been proposed that the maturase activity of LAGLIDADG enzymes could evolve when opportunities to promote intron mobility are limited or non-existent because all potential target sites within a population are saturated with introns [3,4]. In this case, purifying selection on LAGLIDADG coding regions would be relaxed, allowing higher rates of non-synonymous substitutions leading to amino acid replacements. Variants of the motif with RNA maturase activity are not being degraded and eventually deleted [3,4,60]. Interestingly, we found that all LAGLIDADG enzymes in complex Scleractinia and Corallimorpharia are highly degraded. In addition, complex Scleractinia lacks the catalytic metal-binding acidic residues (D or E), while they are poorly conserved in Corallimorpharia. Thus, they might lack endonuclease activity or use a different mechanism for DNA hydrolysis than canonical enzymes. A further possibility is that HEs have switched to another function, for instance, acting as RNA maturase [3]. This might be consistent with the fact that all members of Corallimorpharia and one of the clusters of complex Scleractinia (i.e., the cluster containing *Porites rus*) possess intron 884. This indicates that the HEGs are probably within the extinction phase of the intron/endonuclease life cycle [15,22]. Thus, we suggest that group I introns and their associated HEGs experience a cycle of invasion, mutational degradation of its form and function, and deletion from the host genome, re-establishing the target sequence [61]. This hypothesis is supported by the shorter size of the homing endonuclease observed in complex Scleractinia and Corallimorpharia in comparison to robust Scleractinia (Figs 5 and 6). The size of the coding region serves as evidence for HEG degradation, indicating that shorter HEGs are placed in advanced stages of the intron degradation cycle as previously shown for sea anemones [23].

The putative degradation of LAGLIDADG motifs has profound implications for intron mobility. On the one hand, endonucleases with conserved LAGLIDADG motifs are potentially capable of invading intron-less alleles via horizontal transfer. On the other hand, degraded endonucleases most likely do no longer function as site-specific endonucleases. In that case, the intron/HEG transmission depends largely on vertical transmission via mitochondrial maternal inheritance. We propose that a different functionality of LAGLIDADG motifs accounts for the two patterns of intron distribution observed in our phylogeny: a probably single origin of intron 884 in complex Scleractinia and Corallimorpharia, together with a lack of endonuclease activity, and at least five independent origins of intron 720 in robust Scleractinia due to a functional endonuclease. Given the highly conserved endonuclease, intron 720 in robust Scleractinia has the potential of being transmitted horizontally among populations and across species. This is consistent with the idea that frequent horizontal transmission is necessary for the long-term persistence of homing endonuclease genes [15]. In contrast, the evolutionary history of intron 884 in complex Scleractinia and Corallimorpharia is largely driven by vertical inheritance, due to the degraded homing endonuclease that limits intron mobility.

Finally, the homology of the HEGs was validated through the comparative analyses of secondary and tertiary protein structures, which allow the identification of homologous regions in spite of differences found at the primary sequence level [37,47]. The key principles for such analyses are: (i) the protein structure is assumed to be more conserved than the protein sequence, and (ii) only a finite and relatively small (1,000–10,000) number of unique protein folds occur in nature [62].

### More recent intron gains in robust Scleractinia than in complex Scleractinia and Corallimorpharia

The completion of the intron degradation cycle requires a certain amount of time as shown for some cnidarians in which a rate of one cycle per 100 my may be sufficient to allow invasion, cycling, and persistence of HEGs [22]. The evolutionary history of Scleractinia and Corallimorpharia covers a time frame sufficiently long to permit intron invasion, fixation, and posterior degradation. However, previous divergence time estimates of Scleractinia differ considerably depending on the genes used. Time-calibrated phylogenies based on mitochondrial 12S and 16S rRNAs, and on the nuclear 28S rRNA suggest an age for the MRCA of robust/complex Scleractinia of about 415 my [63], whereas another study based on *COX*I/*cyt b* genes and a more sophisticated calibration approach indicates a considerably younger age (250 mya) [53]. As we used the same calibration strategy as in the latter study, our time estimates are very similar, suggesting that the split of robust and complex Scleractinia occurred c. 260 mya, i.e., shortly before the onset of a massive coral diversification during the mid-Triassic (250 mya) [64–66]. The long time frame together with conserved target sequences may have provided repeated opportunities for intron homing in Scleractinia and Corallimorpharia. Thus, the presence of introns/HEGs in different stages of the intron cycle can be expected. The high conservation of the potential target sequences for the endonuclease may also be explained by a low mitochondrial substitution rate of only 0.03% per my [67] for scleractinian mitochondrial genomes [32,33,35].

The observed patterns of intron distribution and the ancestral intron type inference indicate at least five independent intron gains in robust Scleractinia during the last c. 31 my. In contrast, the number and timing of intron 884 acquisitions in complex Scleractinia and Corallimorpharia remains elusive. However, considering the intron life cycle framework, we propose that intron 884 was inserted early in the evolutionary history of cnidarians, and then rapidly invaded the available intron-less alleles via horizontal transfer. After fixation, the selective pressure for maintaining endonuclease activity decreased, finally leading to the degradation of the HEG as previously shown for sea anemones [23]. Given these findings, we hypothesize that intron 720, which so far has not been reported in complex Scleractinia, may potentially appear in species of this group. In contrast, intron 884 will probably remain absent in robust Scleractinia due to its non-functional endonucleases.

### Intron occurrence in Scleractinia and Corallimorpharia: Biogeographic implications

Our phylogeny (Figs 5 and 6) revealed a distinct geographic pattern of intron type occurrence. Taxa containing intron 720 are only found in the Indo-Pacific Ocean (see also [29]), whereas intron 884 is found in species inhabiting both the Indo-Pacific and the Atlantic Ocean/Mediterranean. Evidence from recent models of coral reef biodiversity dynamics [68] and from the coral fossil record [69] suggest that the location of the major marine biodiversity hotspot has moved across the globe during the last 50 my. During the mid/late Eocene—Oligocene (35–40 mya), the Tethys closure was associated with a narrowing coastal corridor that allowed a faunal migration of Tethys elements into the Indo-Australian Archipelago [68]. Thus, we propose that intron 720 was probably transferred from a still unknown donor to robust Scleractinia during the Tethys closure, isolating the intron-containing taxa from their Atlantic counterparts, and preventing opportunities for intron 720 transfer. Likewise, the cosmopolitan geographic occurrence of taxa containing intron 884 might be explained by the earlier insertion event.

## Conclusions

This study represents the first assessment of the role of the LAGLIDADG motifs during the evolutionary history of group I introns and homing endonuclease genes in the orders Scleractinia and Corallimorpharia. We showed that the LAGLIDADG motifs of complex Scleractinia and Corallimorpharia are degraded, with a lack of the conserved acidic residues necessary for hydrolysis of the DNA backbone of the target sequence. In contrast, both motifs are well defined in robust Scleractinia. Therefore, we suggest that vertical inheritance of group I introns due to non-functional homing explains intron occurrence in complex Scleractinia and Corallimorpharia, whereas both horizontal and vertical transfer gave rise to the present intron distribution in robust Scleractinia. Molecular-clock analyses and ancestral intron type inference indicated at least five independent intron gains in robust Scleractinia during the last c. 31 my. Timing and number of intron gains for complex Scleractinia and Corallimorpharia, however, remain elusive. We further hypothesize that the timing of intron 720 transfer to robust Scleractinia corresponds to the evolution of the Tethys Sea, isolating the intron-containing taxa from their Atlantic counterparts. In summary, our data suggest a complex evolutionary history of introns in Scleractinia and Corallimorpharia mainly driven by endonuclease functionality.

## Supporting information

S1 Fig Intron alignment found in complex Scleractinia using secondary structure elements. Secondary structures of intron 884 of complex Scleractinia are similar based on the number of loop and stem regions (P1-P9) of canonical group I intron structures. The only exception was found in *Siderastrea radians* intron, which differs in the P5 region. Boxes indicate LAGLIDADG ORF start and stop codons, which are found in frame with the intron sequence.
(TIF)

S2 Fig Schematic representation of selected *COXI* exons of complex Scleractinia (blue bar), robust Scleractinia (green bar), and Corallimorpharia (red bar). Boxes show insertion position of intron 720 (orange) and 884 (purple). Symbols (+) and (-) in the insertion sites boxes indicate intron presence or absence, respectively. Shaded areas represent conserved sequences. N indicates no available sequences for those sites. Due to space constraints, only a small number of *COXI* sequences is portrayed here.
(TIF)

S3 Fig Evolutionary rates of the *COXI* gene and of the LAGLIDADG homing endonuclease gene in robust and complex Scleractinia. The rate of nucleotide substitutions causing amino acid changes (K_a_) is plotted relative to substitutions at silent sites (K_s_). Circles represent K_a_/K_s_ ratios. The dotted line indicates the theoretical expectation of neutral evolution (K_a_/K_s_ = 1). The area below the dotted line represents purifying selection (K_a_<K_s_).
(TIF)

S4 Fig Alignment of the putative LAGLIDADG motifs found in Scleractinia and Corallimorpharia. Both motifs are well conserved in robust Scleractinia, whereas complex Scleractinia and Corallimorpharia displayed degraded motifs.
(TIF)

S1 Table Taxonomic information, accession numbers, and intron insertion sites of the intron-containing taxa studied. *Insertion site based on the human *COXI* gene as a counting reference. **The *COXI* gene of the sponge *P*. cf. *onkodes* has two introns in positions 720 and 711. Intron 711 does not contain any ORF. ***Excluded from the molecular-clock analysis due to missing data for *cyt b*.
(DOC)

S2 Table GenBank accession numbers for the *COXI* and *cyt b* genes used for the divergence time estimations. Taxa are sorted alphabetically.
(DOC)
