# Supplementary figures and images for: Evolutionary and biogeographical implications of degraded LAGLIDADG endonuclease functionality and group I intron occurrence in stony corals (Scleractinia) and mushroom corals (Corallimorpharia)
Source: PLoS One. 2017 Mar 9;12(3):e0173734. doi: 10.1371/journal.pone.0173734 (PMC5344465; doi:10.1371/journal.pone.0173734)

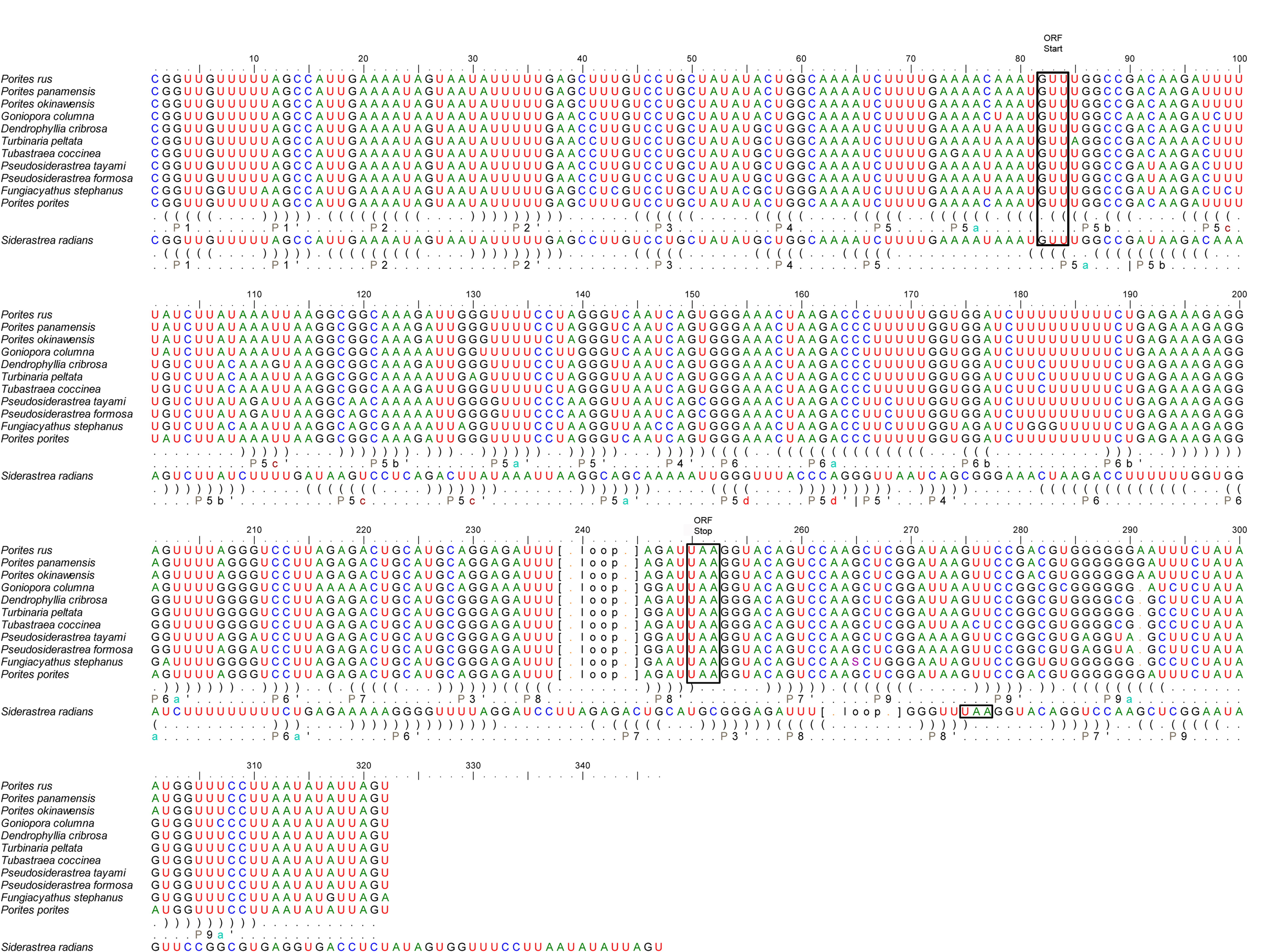

Supplement: S1 Fig — Secondary structures of intron 884 of complex Scleractinia are similar based on the number of loop and stem regions (P1-P9) of canonical group I intron structures. The only exception was found in Siderastrea radians intron, which differs in the P5 region. Boxes indicate LAGLIDADG ORF start and stop codons, which are found in frame with the intron sequence. (TIF) [file pone.0173734.s001.tif]

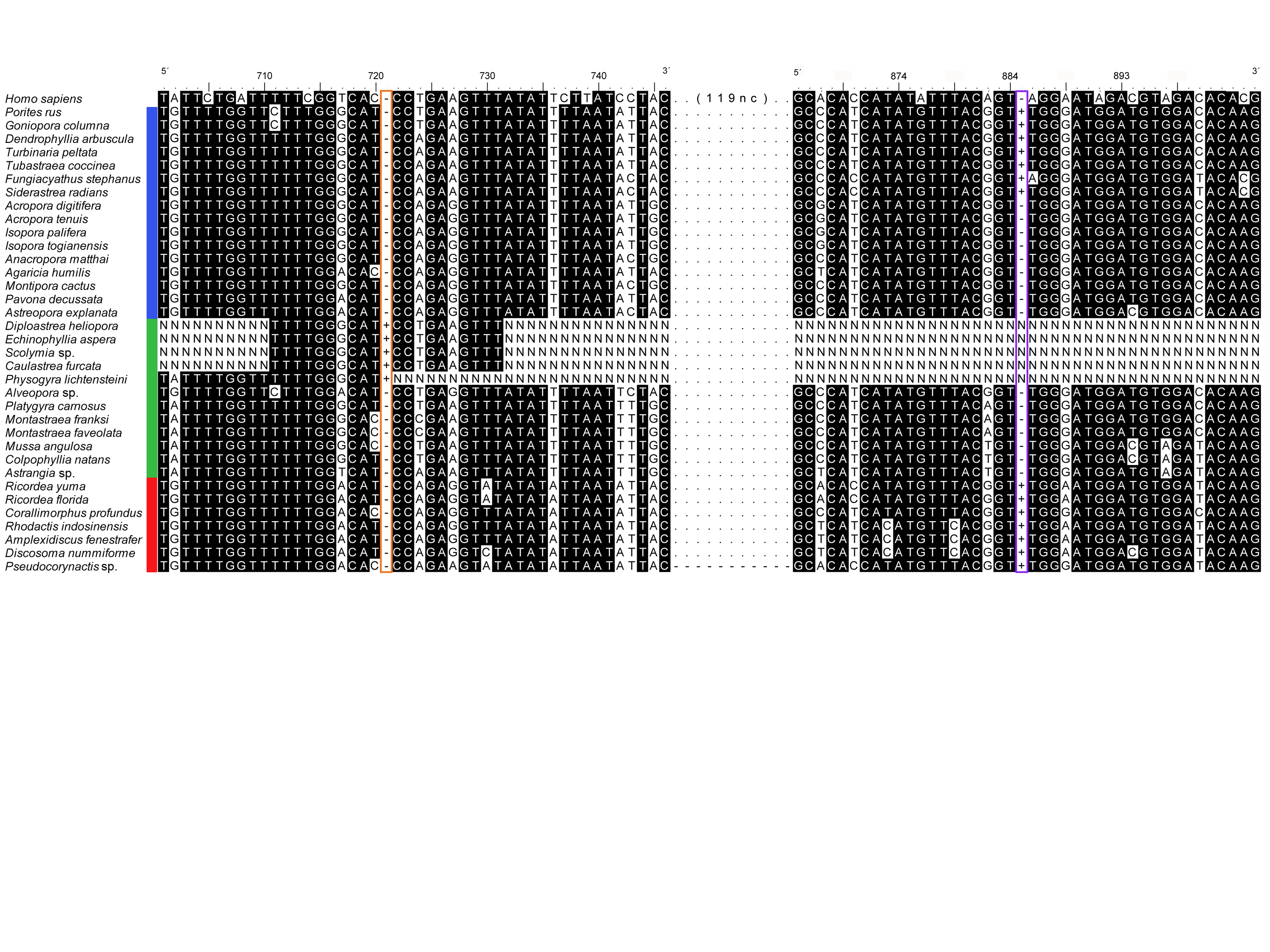

Supplement: S2 Fig — Boxes show insertion position of intron 720 (orange) and 884 (purple). Symbols (+) and (-) in the insertion sites boxes indicate intron presence or absence, respectively. Shaded areas represent conserved sequences. N indicates no available sequences for those sites. Due to space constraints, only a small number of COXI sequences is portrayed here. (TIF) [file pone.0173734.s002.tif]

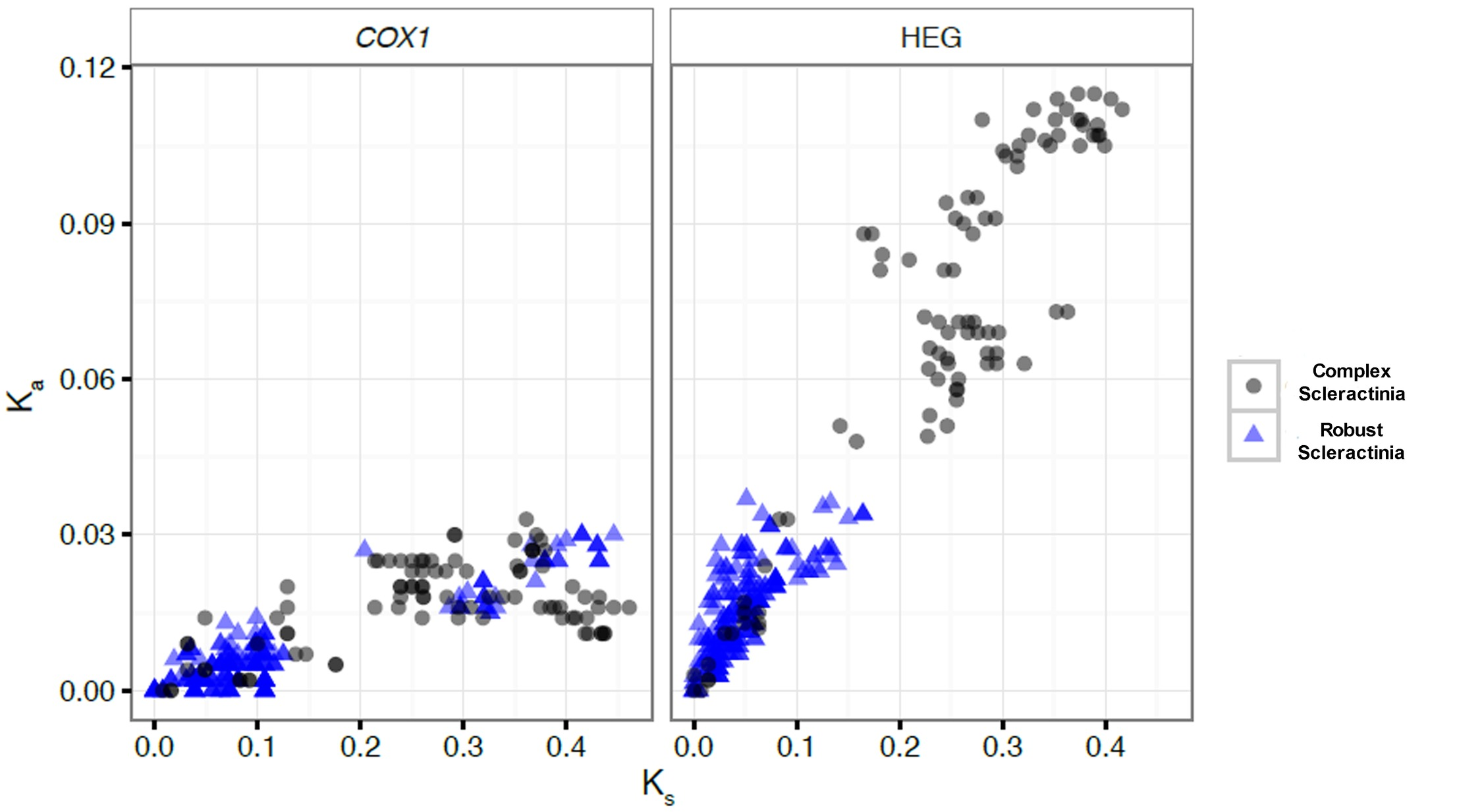

Supplement: S3 Fig — The rate of nucleotide substitutions causing amino acid changes (Ka) is plotted relative to substitutions at silent sites (Ks). Circles represent Ka/Ks ratios. The dotted line indicates the theoretical expectation of neutral evolution (Ka/Ks = 1). The area below the dotted line represents purifying selection (Ka<Ks). (TIF) [file pone.0173734.s003.tif]

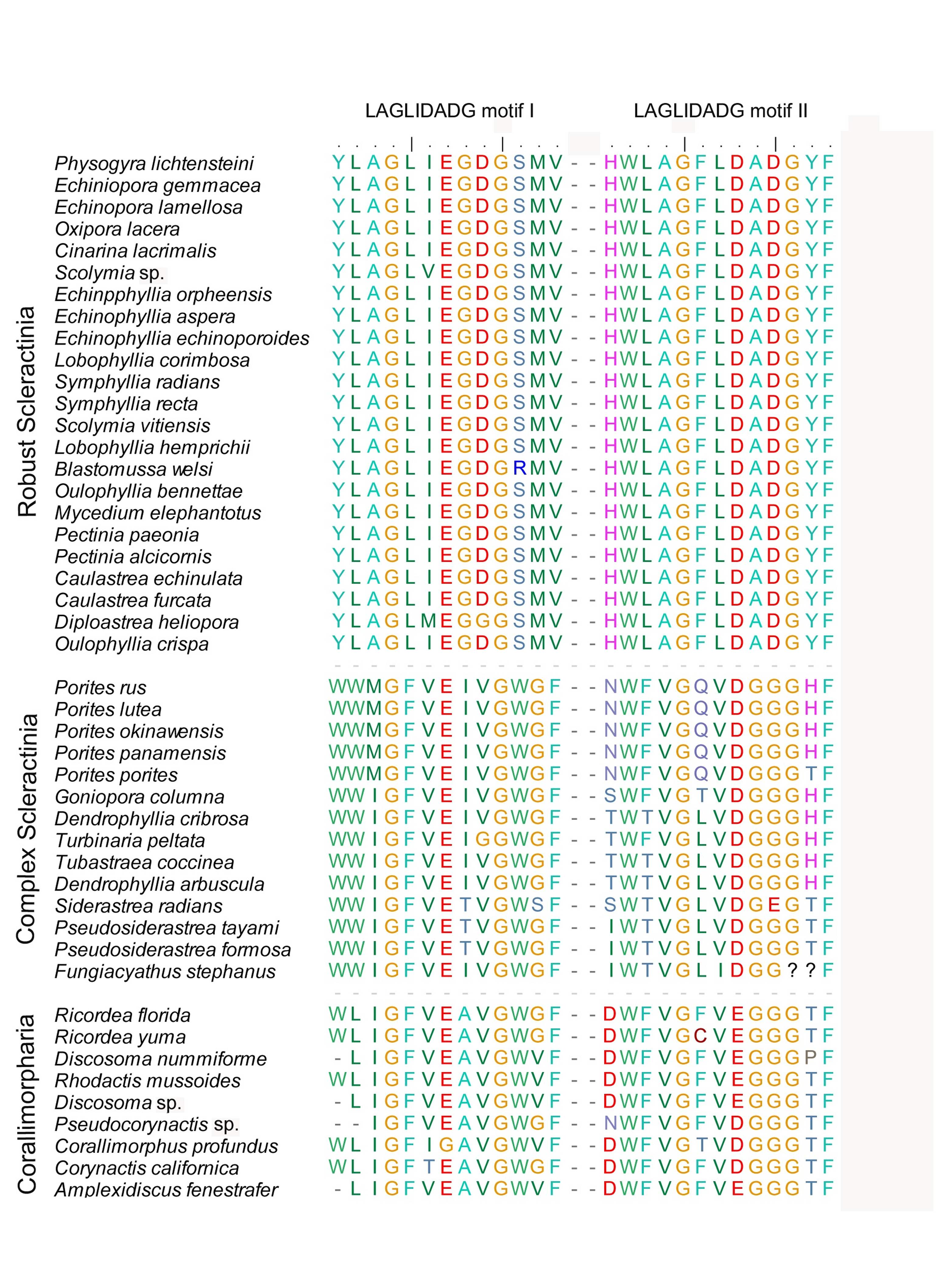

Supplement: S4 Fig — Both motifs are well conserved in robust Scleractinia, whereas complex Scleractinia and Corallimorpharia displayed degraded motifs. (TIF) [file pone.0173734.s004.tif]
