# Supplementary material for: Evolutionary and biogeographical implications of degraded LAGLIDADG endonuclease functionality and group I intron occurrence in stony corals (Scleractinia) and mushroom corals (Corallimorpharia)
Source: PLoS One. 2017 Mar 9;12(3):e0173734. doi: 10.1371/journal.pone.0173734 (PMC5344465; doi:10.1371/journal.pone.0173734)
Supplement: S1 Table — *Insertion site based on the human COXI gene as a counting reference. **The COXI gene of the sponge P. cf. onkodes has two introns in positions 720 and 711. Intron 711 does not contain any ORF. ***Excluded from the molecular-clock analysis due to missing data for cyt b. (DOC) [file pone.0173734.s005.doc]

**S1 Table. Taxonomic information, accession numbers, and intron insertion sites of the intron-containing taxa studied.**

| **Group** | **Species** | **GenBank accession number** | **Intron insertion site*** |
| --- | --- | --- | --- |
| Robust Scleractinia | *Physogyra lichtensteini* | AB289562 | 720 |
|  | *Echinopora lamellosa* | FJ345449 |  |
|  | *Echinopora gemmacea* | FJ345448 |  |
|  | *Oxipora lacera* | AB289571 |  |
|  | *Cinarina lacrymalis* | AB289568 |  |
|  | *Scolymia* sp. | AB289570 |  |
|  | *Echinophyllia orpheensis* | AB289574 |  |
|  | *Echinophyllia aspera* | AB289572 |  |
|  | *Echinophyllia echinoporoides* | AB289573 |  |
|  | *Lobophyllia corimbosa* | AB289576 |  |
|  | *Symphyllia radians* | AB289578 |  |
|  | *Symphyllia recta* | AB289577 |  |
|  | *Lobophyllia hemprichii* | AB289575 |  |
|  | *Scolymia vitiensis* | AB289569 |  |
|  | *Blastomussa welsi* | AB289563 |  |
|  | *Oulophyllia bennettae* | AB289581 |  |
|  | *Mycedium elephantotus* | HEAB289582 |  |
|  | *Pectinia paeonia* | AB289584 |  |
|  | *Pectinia alcicornis* | AB289583 |  |
|  | *Caulastrea echinulata**** | FJ345445 |  |
|  | *Caulastrea furcata* | AB289579 |  |
|  | *Diploastrea heliopora* | AB289567 |  |
|  | *Oulophyllia crispa* | AB289580 |  |
| Complex Scleractinia | *Porites lutea* | KU159432 | 884 |
|  | *Porites okinawensis* | NC_015644 |  |
|  | *Porites panamensis* | NC_024182 |  |
|  | *Porites porites* | NC_008166 |  |
|  | *Porites rus* | LN864762 |  |
|  | *Goniopor columna* | NC_015643 |  |
|  | *Turbinaria peltata* | NC_024671 |  |
|  | *Dendrophyllia arbuscula* | NC_027590 |  |
|  | *Dendrophyllia cribrosa* | NC_026026 |  |
|  | *Tubastraea coccinea* | NC_026025 |  |
|  | *Fungiacyathus stephanus* | NC_015640 |  |
|  | *Siderastraea radians* | NC_008167 |  |
|  | *Pseudosiderastrea formosa* | NC_026530 |  |
|  | *Pseudosiderastrea tayami* | NC_026531 |  |
| Corallimorpharia | *Ricordea florida* | NC_008159 | 884 |
|  | *Ricorde yuma* | NC_027106 |  |
|  | *Discosoma nummiforme* | KP938434 |  |
|  | *Rhodactis mussoides* | KP938439. |  |
|  | *Discosoma* sp. | DQ643966 |  |
|  | *Pseudocorynactis* sp. | KP938437 |  |
|  | *Corallimorphus profundus* | KP938440 |  |
|  | *Corynactis californica* | NC_027102 |  |
|  | *Amplexidiscus fenestrafer* | NC_027101 |  |
|  | *Rhodactis indosinensis* | NC_027103 |  |
|  | *Rhodactis s*p. | DQ640647 |  |
| Actiniaria | *Metridium senile* | HG423143 | 884 |
|  | *Aiptasia pulchella* | NC_022265 |  |
|  | *Hormatia digitata* | NC_022471 |  |
|  | *Bolocera tuediae* | NC_022470 |  |
|  | *Urticina eques* | NC_022469 |  |
| Antipatharia | *Stichopathes lutkeni* | NC_018377 | 884 |
| Zoantharia | *Savalia savaglia* | NC_008827 | 867 |
| Porifera | *Plakinastrella* cf. *onkodes* | NC_010217 | 720** |

*Insertion site based on the human *COXI* gene as a counting reference.

**The *COXI* gene of the sponge *P. onkodes* has two introns in positions 720 and 711. Intron 711 does not contain any ORF.

***Excluded from the molecular-clock analysis due to missing data for *cyt b.*
