# Supplementary material for: Evolutionary and biogeographical implications of degraded LAGLIDADG endonuclease functionality and group I intron occurrence in stony corals (Scleractinia) and mushroom corals (Corallimorpharia)
Source: PLoS One. 2017 Mar 9;12(3):e0173734. doi: 10.1371/journal.pone.0173734 (PMC5344465; doi:10.1371/journal.pone.0173734)
Supplement: S2 Table — Taxa are sorted alphabetically. (DOC) [file pone.0173734.s006.doc]

**S2 Table.** **GenBank accession numbers for the *COXI* and *cyt b* genes used for the divergence time estimations.** Taxa are sorted alphabetically.

|  | **GenBank accession numbers** | |
| --- | --- | --- |
| **Species** | ***COXI*** | ***cyt b*** |
|  |  |  |
| *Acanella eburnea* | EF672731 | EF672731 |
| *Acanthastrea echinata* | LN875928 | AB117327 |
| *Acanthastrea hillae* | LN875878 | AB441284 |
| *Acanthastrea rotundata* | AB117251 | AB117328 |
| *Acropora digitifera* | KF448535 | KF448535 |
| *Acropora florida* | KF448533 | KF448533 |
| *Acropora tenuis* | AF338425 | AB033181 |
| *Agaricia fragilis* | KM051016 | KM051016 |
| *Agaricia humilis* | AB441219 | DQ643831 |
| *Aiptasia pulchella* | HG423148 | HG423148 |
| *Alveopora* sp. | AB907091 | KJ634271 |
| *Amplexidiscus fenestrafer* | KP938435 | KP938435 |
| *Anacropora forbesi* | AB441251 | AB441336 |
| *Anacropora matthai* | AB441250 | AB441335 |
| *Astrangia* sp. | DQ643832 | DQ643832 |
| *Astreopora explanata* | KJ634269 | KJ634269 |
| *Astreopora myriophthalma* | KJ634272 | KJ634272 |
| *Barabattoia amicorum* | AB441193 | AB441278 |
| *Blastomussa wellsi* | AB289563 | AB289565 |
| *Bolocera tuediae* | HG423145 | HG423145 |
| *Caulastraea furcata* | AB117274 | AB117355 |
| *Chrysophates formosa* | JX560754 | DQ304771 |
| *Cladocora arbuscula* | AB117292 | AB117377 |
| *Colpophyllia natans* | AY451346 | DQ643833 |
| *Corallimorphus profundus* | KP938440 | KP938440 |
| *Corynactis californica* | KP938436 | KP938436 |
| *Coscinaraea columna* | AB441210 | AB441295 |
| *Ctenella chagius* | AB441208 | AB441293 |
| *Cynarina lacrymalis* | AB117246 | AB117323 |
| *Cyphastrea chalcidicum* | FJ345415 | AB117336 |
| *Cyphastrea serailia* | AB117258 | AB117334 |
| *Dendrogyra cylindrus* | AB117299 | AB117384 |
| *Dendrophyllia arbuscula* | KR824937 | KR824937 |
| *Dendrophyllia cribrosa* | JQ290080 | JQ290080 |
| *Dendrophyllia* sp. | AB441239 | AB441324 |
| *Dichocoenia stokesi* | AY451360 | AB117383 |
| *Diploastrea heliopora* | EU371660 | AB117375 |
| *Diploria clivosa* | AB117226 | AB117304 |
| *Diploria labyrinthiformis* | AY451348 | AB117302 |
| *Diploria strigosa* | AY451349 | AB117303 |
| *Discosoma carlgreni* | AB441268 | AB441353 |
| *Discosoma nummiforme* | KP938434 | KP938434 |
| *Discosoma* sp. | AB441270 | DQ643966 |
| *Echinophyllia aspera* | HE654648 | AB117329 |
| *Echinophyllia echinoporoides* | AB289573 | AB117331 |
| *Echinophyllia orpheensis* | LN999911 | AB117330 |
| *Echinopora gemmacea* | FJ345418 | AB117342 |
| *Echinopora lamellosa* | FJ345419 | JQ966169 |
| *Echinopora pacificus* | AB117262 | AB117340 |
| *Euphyllia ancora* | AB441204 | JF825139 |
| *Euphyllia divisa* | AB441203 | AB441288 |
| *Euphyllia glabrescens* | AB441206 | AB441291 |
| *Eusmilia fastigiata* | AB117294 | AB117380 |
| *Favia favus* | HQ203257 | AB117346 |
| *Favia fragum* | AY451350 | AB117300 |
| *Favia leptophylla* | AB117230 | AB117307 |
| *Favia pallida* | EU371676 | AB117345 |
| *Favia speciosa* | AB441194 | AB441279 |
| *Favia stelligera* | HQ203265 | AB117343 |
| *Favites chinensis* | HQ203268 | AB117349 |
| *Favites halicora* | HE654590 | AB117347 |
| *Fungiacyathus* sp. | AB441255 | AB441340 |
| *Fungiacyathus stephanus* | JF825138 | JF825138 |
| *Galaxea fascicularis* | AB441201 | KU159433 |
| *Gardineroseris planulata* | AB441218 | AB441303 |
| *Goniastrea aspera* | AB117271 | AB117351 |
| *Goniastrea pectinata* | FJ345434 | AB117350 |
| *Goniopora columna* | AB907032 | JF825141 |
| *Goniopora* sp. | AB441241 | AB441326 |
| *Helioseris cucullata* | AB441220 | AB441305 |
| *Herpolitha limax* | AB441223 | AB441308 |
| *Hormathia digitata* | HG423146 | HG423146 |
| *Hydnophora exesa* | HE654621 | AB117370 |
| *Hydnophora grandis* | AB117286 | AB117371 |
| *Isophyllia sinuosa* | AB117238 | AB117315 |
| *Isopora brueggemanni* | AB441247 | AB441332 |
| *Isopora palifera* | KJ634270 | KJ634270 |
| *Isopora togianensis* | KJ634268 | KJ634268 |
| *Leptastrea pruinosa* | AB441196 | AB441281 |
| *Leptoria irregularis* | HQ203279 | AB117352 |
| *Leptoria phrygia* | HE654611 | AB117354 |
| *Lobactis scutaria* | HM048841 | AB441309 |
| *Lobophyllia corymbosa* | AB117241 | AB117318 |
| *Lobophyllia hemprichii* | AB117240 | AB117317 |
| *Lobophyllia pachysepta* | LN999910 | AB117319 |
| *Lophelia pertusa* | FR821799 | FR821799 |
| *Madracis mirabilis* | EU400212 | EU400212 |
| *Madrepora oculata* | JX236041 | JX236041 |
| *Manicina areolata* | AB117227 | AB117305 |
| *Meandrina meandrites* | AB117295 | AB117381 |
| *Merulina ampliata* | HQ203280 | AB117368 |
| *Merulina scabricula* | HQ203281 | AB117369 |
| *Metridium senile* | HG423143 | HG423143 |
| *Micromussa amakusensis* | LN875883 | AB441285 |
| *Montastrea annularis* | AF013737 | AP008974 |
| *Montastrea cavernosa* | AF108712 | AB117373 |
| *Montastrea curta* | EU371706 | AB117359 |
| *Montastrea faveolata* | AP008978 | AP008978 |
| *Montastrea franksi* | AP008976 | AP008975 |
| *Montastrea magnistellata* | HQ203287 | AB117360 |
| *Montastrea valenciennesi* | EU371719 | AB117361 |
| *Montipora cactus* | AB441252 | AB441337 |
| *Mussa angulosa* | DQ643834 | DQ643834 |
| *Mussismilia braziliensis* | AB117231 | AB117309 |
| *Mussismilia harttii* | AB117232 | AB117308 |
| *Mussismilia hispida* | AB117233 | AB117310 |
| *Mycedium elephantotus* | HQ203294 | AB117367 |
| *Mycetophyllia aliciae* | AY451364 | AB117312 |
| *Mycetophyllia daniana* | AB117234 | AB117311 |
| *Oculina diffusa* | AB117293 | AB117379 |
| *Oulastrea crispata* | FJ345435 | AB441282 |
| *Oulophyllia bennettae* | FJ345438 | AB117358 |
| *Oulophyllia crispa* | AB117276 | AB117357 |
| *Oxypora lacera* | HF954227 | AB117332 |
| *Pachyseris speciosa* | AB441222 | AB441307 |
| *Paragoniastrea deformis* | AB441195 | AB441280 |
| *Pavona clavus* | DQ643836 | DQ643836 |
| *Pavona decussata* | KP231535 | KP231535 |
| *Pectinia alcicornis* | AB117385 | AB117364 |
| *Pectinia paeonia* | HQ203301 | AB117365 |
| *Physogyra lichtensteini* | HF954195 | AB289564 |
| *Plakinastrella* cf. *onkodes* | EU237487 | EU237487 |
| *Platygyra carnosus* | JX911333 | JX911333 |
| *Platygyra daedalea* | HE654617 | AB117362 |
| *Platygyra lamellina* | HQ203302 | AB117363 |
| *Plesiastrea versipora* | FR837984 | AB289566 |
| *Pocillopora damicornis* | KF194194 | EU400213 |
| *Pocillopora eydouxi* | EF526303 | EF526303 |
| *Pocillopora verrucosa* | AB441230 | AB441315 |
| *Polycyathus* sp. | JF825140 | JF82514 |
| *Porites astreoides* | FJ423961 | AB441327 |
| *Porites lutea* | KF271436 | AB441328 |
| *Porites okinawensis* | JF825142. | JF825142 |
| *Porites panamensis* | KJ546638 | KJ546638 |
| *Porites porites* | DQ643837 | DQ643837 |
| *Porites rus* | LN864762 | LN864762 |
| *Psammocora contigua* | AB441209 | AB441294 |
| *Pseudocorynactis* sp. | KP938437 | KP938437 |
| *Pseudosiderastrea formosa* | KP260632 | KP260632 |
| *Pseudosiderastrea tayami* | KP260633 | KP260633 |
| *Rhodactis indosinensis* | KP938438 | KP938438 |
| *Rhodactis mussoides* | KP938439 | KP938439 |
| *Rhodactis* sp. | AB441265 | DQ640647 |
| *Ricordea florida* | NC_008159.1 | NC_008159.1 |
| *Ricordea yuma* | KP938441 | KP938441 |
| *Savalia savaglia* | HQ110947 | DQ825686 |
| *Scapophyllia cylindrica* | FJ345444 | AB441283 |
| *Scolymia cubensis* | AB117237 | AB117313 |
| *Scolymia* sp. | AB117248 | AB117325 |
| *Scolymia vitiensis* | LK022358 | AB117324 |
| *Seriatopora caliendrum* | EF633601 | EF633601 |
| *Seriatopora hystrix* | AB441234 | AB441319 |
| *Seriatopora* sp. | AB441232 | AB441317 |
| *Siderastrea radians* | KM391401 | AB441297 |
| *Siderastrea savignyana* | AB441214 | AB441299 |
| *Siderastrea siderea* | AB441211 | AB441296 |
| *Siderastrea stellata* | AB441213 | AB441298 |
| *Solenastrea bournoni* | AY451359 | AB117376 |
| *Solenosmilia variabilis* | KM609294 | KM609294 |
| *Stephanocoenia michelinii* | AB441228 | AB441313 |
| *Stichopathes lutkeni* | NC_018377 | NC_018377 |
| *Stylocoeniella guentheri* | AB441225 | AB44131 |
| *Stylophora pistillata* | AB441231 | EU400214 |
| *Symphyllia agaricia* | HF954263 | AB117320 |
| *Symphyllia radians* | HF954266 | AB117322 |
| *Symphyllia recta* | AB117244 | AB117321 |
| *Trachyphyllia geoffroyi* | AB117287 | AB117372 |
| *Tubastraea aurea* | AB441235 | AB441322 |
| *Tubastraea coccinea* | JQ290078 | JQ290078 |
| *Turbinaria peltata* | HG965364 | KJ725201 |
| *Urticina eques* | HG423144 | HG423144 |
